# Hypertriglyceridemic-waist phenotype is strongly associated with cardiovascular risk factor clustering in Chinese adolescents

**DOI:** 10.1038/s41598-022-19690-8

**Published:** 2022-09-14

**Authors:** Rongrong Cai, Jinyu Zhou, Ling Bai, Yangyang Dong, Wenqing Ding

**Affiliations:** 1 School of Public Health and Management, Ningxia Medical University, Yinchuan, China; 2 Key Laboratory of Environmental Factors and Chronic Disease Control, Ningxia Medical University, No.1160, Shengli Street, Xingqing District, Yinchuan, Ningxia China

**Keywords:** Epidemiology, Paediatric research

## Abstract

The early identification of predictors related to cardiovascular risk factor clustering (CVRFC) in adolescents can help prevent Cardiovascular disease. The hypertriglyceridemic-waist circumference (HTW) phenotype is considered a simple and useful indicator to identify cardiovascular disease. However, there is limited research on the relationship between the HTW phenotype and (CVRFC) in adolescents. It is unclear whether the HTW phenotype can identify early the risk of developing CVRFC in adolescents. The study aimed to examine the association of the HTW phenotype with CVRFC in adolescents. A total of 1478 adolescents aged 12–18 years were classified into normal waist circumference (WC) and normal triglyceride (TG) (NWNT, 66.4%), normal WC and high TG (HTG, 5.5%), enlarged WC and normal TG (EW, 22.2%) and enlarged WC and high TG (HTW, 5.8%). High TG was defined as TG ≥ 1.47 mmol/L and enlarged WC ≥ 90th percentile by gender and age. CVRFs in this study included elevated blood pressure (BP), impaired fasting glucose (IFG), high total cholesterol (TC), low high-density lipoprotein cholesterol (HDL-C), and high low-density lipoprotein cholesterol (LDL-C). CVRFC ≥ 2 or CVRFC ≥ 3 were defined as the presence of at least two or three cardiovascular risk factors. After adjustment for BMI, gender and age, the HTW phenotype increased the risk of CVRFC ≥ 2 and CVRFC ≥ 3 compared to the NTNW phenotype, OR and 95%CI were 2.40 (1.23–4.58) and 3.63 (1.49–8.86), respectively. After stratification by gender, similar results were found in boys, however, girls with the EW phenotype had a lower risk of CVRFC ≥ 2 and CVRFC ≥ 3 compared with the NTNW phenotype after adjustment for BMI and age. The area under the ROC curve was 0.698 (0.661–0.736) and 0.782 (0.725–0.840) when TG was combined with WC to detect cardiovascular risk factors clustering, which was better than BMI, WHtR, TG or WC alone. And similar results were obtained for both boys and girls when stratified by gender. These results revealed that different combinations of TG and WC levels are closely associated with cardiovascular risk factors clustering in both boys and girls, and TG combining WC performed better than BMI, WHtR, TG or WC alone in detecting cardiovascular risk factor clustering in adolescents.

## Introduction

Cardiovascular disease (CVD) is currently the leading cause of death worldwide and contributes to millions of deaths and disability-adjusted life-years lost^1,2^. It has been shown that cardiovascular risk factors (CVRFs) in childhood are strongly associated with CVD in adulthood^3,4^. CVRFs mainly include elevated blood pressure (BP), hyperglycemia and dyslipidemia. Previous studies found that the prevalence of cardiovascular risk factor clustering (CVRFC) has increased in childhood and adolescents^5^. The clustering of cardiovascular risk factors in youngsters is known to be associated with accelerated atherogenesis and increased risk of many CVD such as type 2 diabetes mellitus (T2DM) and hypertension in adulthood^6,7^. Therefore, the early identification of factors associated with CVRFs and CVRFC may be vital for preventing these diseases, particularly for adolescents.

Hypertriglyceridemic-waist (HTW) phenotype proposed by Lemieux et al. combines the lipid indicator triglyceride (TG) with the obesity indicator waist circumference (WC), which are considered a simple proxy for abdominal obesity and metabolic dysfunction^8^. Previous studies have shown that the HTW phenotype is strongly associated with CVD, and some adult studies have concluded that the HTW phenotype is a simple and useful marker that can be used to identify CVD^9–12^. Because of the low prevalence of CVD in adolescents, it is difficult to directly study the association between different HTW phenotypes and the risk of CVD in adolescents. Cardiovascular risk factors and cardiovascular risk factor clustering, as early signals of CVD, if these signals can be detected in adolescents and taken appropriate measures, it will be important to the prevention and treatment of CVD. However, the association between the HTW phenotype and cardiovascular risk factors and cardiovascular risk factor clustering in adolescents is unclear and relevant research is limited.

Therefore, this study investigates the relationship between different HTW phenotypes and cardiovascular risk factors and cardiovascular risk factors clustering in urban adolescents in China, to provide a scientific basis for the prevention and intervention of cardiovascular disease in adolescents.

## Methods

### Study participants

The sample size was determined according to the cross-sectional design, and the calculation formula was $n=\frac{{\mu }_{\alpha /2}^{2} p(1-p)}{{\delta }^{2}}$, (α = 0.05, δ = 0.02, and *p* is the prevalence of cardiovascular risk factor clustering (CVRFC) ≥ 2). Yan et al. found that the prevalence of CVRFC ≥ 2 was 6.9% and 6.5% in boys and girls, respectively, in Chinese children and adolescents aged 6–18 years^13^. The sample size should be 617 (617 = 1.96^2^ × 0.069 × 0.931/0.02^2^), according to the formula of sample size. Considering the sampling error, the sample size needed to be increased by 5%, so the minimum sample size was 648 (648 = 617 × (1 + 5%)). Data for this study were obtained from a cross-sectional study, using a convenience sampling to select three junior schools and three high schools in Yinchuan city, China, between 2017 and 2020. Using a cluster random sampling method, made a stratification according to the grade level, then classes are randomly selected from each grade (11 junior school classes and 29 high school classes), and finally whole groups of study participants were included in the survey by class. Individuals with physical disabilities, deformities and congenital genetic disorders were excluded. After excluding participants with missing information, a total of 1478 subjects aged 12–18 years were enrolled in this study. All subjects participated in the questionnaire, physical examination and laboratory analysis. All study protocols were approved by the Medical Ethics Review Committee of Ningxia Medical University and all informed consents were acquired from study participants and their guardians (No.2021-G053) and conducted in accordance with the Declaration of Helsinki.

### Physical measurement

Height and weight were measured using a mechanical stadiometer (Model: ZH7082) and an electronic scale (Model: RGT-140), with the subject removing shoes and heavy clothing, both measured twice and averaged for inclusion in the final analysis, to an accuracy of 0.1 cm and 0.1 kg for height and weight respectively. Waist circumference (WC) was measured using a nylon tape measure and the measurements were averaged twice to an accuracy of 0.1 cm. BMI was calculated as weight divided by the squared height (kg/m^2^). Blood pressure (BP) was measured by using a calibrated electronic sphygmomanometer (Model: HEM-7012, Omron, Japan) according to the standard method by the "American Hypertension Education Project Working Group"^14^. A suitable cuff was chosen for the measurement (7 cm, 9 cm, 12 cm, etc. for BP measurement in adolescents) and the subject was seated facing the measurer and BP was measured on the right upper arm with the elbow at the same level as the sphygmomanometer and the heart. Systolic blood pressure (SBP) and diastolic blood pressure (DBP) were measured three times at 1-min intervals, and the average of the last two readings was recorded for the final analysis (a third measurement was taken if the difference between the first two blood pressure values exceeded 10 mm Hg (1 mm Hg = 0.133 kPa)).

### Biochemical analysis

Venous blood samples were collected after at least 12 h of overnight fasting. Fasting plasma glucose (FPG), triglyceride (TG), total cholesterol (TC), high-density lipoprotein cholesterol (HDL-C) and low-density lipoprotein cholesterol (LDL-C) were measured by using an automated biochemistry analyzer (Model: AU480, American). FPG, TG and TC were detected by enzymatic methods, HDL-C and LDL-C were measured by the direct method-peroxidase method.

### Definitions

Cardiovascular risk factors (CVRFs) were defined as follows, elevated WC, elevated BP and impaired fasting glucose (IFG) were defined using the harmonized criteria of the International Diabetes Federation (IDF)^15^: enlarged WC, WC ≥ 90th percentile by gender and age; elevated BP, SBP or DBP ≥ 90th percentile by gender and age; IFG, FPG ≥ 5.6 mmol/L. Adverse lipid concentrations were defined according to the National Heart, Lung, and Blood Institute expert panel on integrated guidelines for cardiovascular health and risk reduction in children and adolescents^16^: high TG, TG ≥ 1.47 mmol/L; high TC, TC ≥ 5.18 mmol/L; high LDL-C, LDL-C ≥ 3.37 mmol/L; low HDL-C, HDL-C ≤ 1.03 mmol/L.

Cardiovascular disease risk factor clustering (CVRFC) refers to the number of five factors as follows: elevated BP, IFG, high TC, high LDL-C and low HDL-C (High TG was not included in the definition of CVRFC in this study to avoid spurious associations). CVRFC ≥ 2 was defined as the presence of at least two cardiovascular risk factors and CVRFC ≥ 3 was defined as the presence of at least three cardiovascular risk factors.

The study subjects were divided into four groups according to WC and TG level:(1) normal TG and normal WC (NWNT): TG < 1.47 mmol/L^16^and WC < 90th percentile by gender and age^15^; (2)hypertriglyceridemia (HTG): TG ≥ 1.47 mmol/L and WC < 90th percentile by gender and age; (3)enlarged WC (EW): TG < 1.47 mmol/L and WC ≥ 90th percentile by gender and age; (4)hypertriglyceridemic-waist (HTW): TG ≥ 1.47 mmol/L and WC ≥ 90th percentile by gender and age.

### Statistical analysis

SPSS 26.0 and GraphPad Prism 7.0 were used for data analysis and mapping. All data were expressed as mean ± standard deviation for continuous variables, *P*_50_(*P*_25_, *P*_75_) for skewed variables and percentages for categorical variables. For comparisons between groups, ANOVA was used for continuous variables, Mann–Whitney U-test for skewed variables and chi-square tests for categorical variables. Height, weight, WC, BMI, SBP and DBP were standardized by ages, Z-score = (measured value—mean by age and gender) / (standard deviation by age and gender). Binary logistic regression analysis was used to analyze the association between different HTW phenotypes and CVRFs, CVRFC ≥ 2 and CVRFC ≥ 3. The covariates included BMI, gender and age. The receiver operating characteristic (ROC) curve was used to compare the effects of BMI, WHtR, TG, WC and TG combining WC for predicting cardiovascular risk factors clustering. Two-sided *P* < 0.05 was regarded as statistically significant.

## Results

### Comparison of the basic characteristics of different HTW phenotypes

A total of 1478 study participants aged 12–18 years were included for analysis. Of these, the NTNW, HTG, EW and HTW phenotypes accounted for 66.4%, 5.5%, 22.2% and 5.8% respectively. Differences in gender, height, height-Zscore, weight, weight-Zscore, BMI, BMI-Zscore, WC, WC-Zscore, WHtR, SBP, SBP-Zscore, DBP, DBP-Zscore, FPG, TG, TC, HDL-C and LDL-C were found among adolescents with different HTW phenotypes (all *P* < 0.01), and individuals with the HTW phenotype had higher height-Zscore, weight-Zscore, BMI, BMI-Zscore, WC, WC-Zscore, WHtR, SBP, SBP-Zscore, DBP, DBP-Zscore and TG than those with the NTNW, HTG and EW phenotypes, respectively (all *P* < 0.05), as shown in Table 1.

**Table 1:** Comparison of basic characteristics of adolescents with different HTW phenotypes.

Variables	NTNW (N = 982)	HTG (N = 82)	EW (N = 328)	HTW (N = 86)	F/H	*P* value
Age (year)	14.8 ± 1.5	14.8 ± 1.6	14.7 ± 1.4	14.6 ± 1.6	0.95	0.414
**Gender**						
Boys N (%)	661 (44.7)	45 (3.0)	149 (10.1)	48 (3.2)	52.19	< 0.001
Girls N (%)	321 (21.7)	37 (2.5)	179 (12.1)	38 (2.6)		
Height (cm)	167.3 ± 8.5	166.1 ± 9.1	169.0 ± 7.8^ab^	169.2 ± 8.2^ab^	5.41	0.001
Height-Z score	− 0.15 ± 0.98	− 0.21 ± 0.94	0.42 ± 0.91^ab^	0.34 ± 0.95^abc^	34.12	< 0.001
Weight (kg)	52.7 ± 7.8	52.4 ± 6.5	70.6 ± 12.5^ab^	75.0 ± 12.7^abc^	411.67	< 0.001
Weight-Z score	− 0.46 ± 0.57	− 0.45 ± 0.49	1.11 ± 0.87^ab^	1.46 ± 0.81^abc^	626.97	< 0.001
BMI (kg/m^2^)	18.8 ± 1.9	19.0 ± 1.93	24.6 ± 3.1^ab^	26.1 ± 3.4^abc^	692.17	< 0.001
BMI-Z score	− 0.47 ± 0.53	− 0.39 ± 0.61	1.10 ± 0.88^ab^	1.54 ± 0.89^abc^	646.81	< 0.001
WC (cm)	68.9 ± 4.4	70.2 ± 3.8^a^	85.7 ± 8.5^ab^	89.4 ± 9.5^abc^	875.65	< 0.001
WC-Z score	− 0.51 ± 0.45	− 0.36 ± 0.45^a^	1.18 ± 0.85^ab^	1.62 ± 0.89^abc^	888.75	< 0.001
WHtR	0.41 ± 0.03	0.42 ± 0.03^a^	0.51 ± 0.04^ab^	0.53 ± 0.05^abc^	838.66	< 0.001
SBP (mm Hg)	109.7 ± 10.4	108.8 ± 11.2	116.2 ± 10.3^ab^	121.2 ± 11.6^abc^	54.84	< 0.001
SBP-Z score	− 0.21 ± 0.91	− 0.28 ± 0.96	0.46 ± 0.96^ab^	0.87 ± 1.01^abc^	71.37	< 0.001
DBP (mm Hg)	66.6 ± 7.7	67.3 ± 6.8	70.0 ± 7.7^ab^	73.9 ± 8.8^abc^	34.42	< 0.001
DBP-Z score	− 0.13 ± 0.96	− 0.11 ± 0.87	0.23 ± 0.97^ab^	0.75 ± 1.12^abc^	30.00	< 0.001
FPG (mmol/L)	4.75 ± 0.68	5.16 ± 1.11^a^	4.72 ± 0.55^b^	4.83 ± 0.68^b^	10.13	< 0.001
TG (mmol/L)*	0.83 (0.67,1.02)	1.67 (1.54,1.91)^a^	0.95 (0.76,1.20)^ab^	1.94 (1.59,2.25)^abc^	483.13	< 0.001
TC (mmol/L)	3.88 ± 0.90	4.65 ± 1.27^a^	3.93 ± 0.82^b^	4.61 ± 1.28^ac^	30.96	< 0.001
HDL-C (mmol/L)	1.48 ± 0.38	1.64 ± 0.61^a^	1.35 ± 0.32^ab^	1.28 ± 0.37^ab^	22.68	< 0.001
LDL-C (mmol/L)	2.08 ± 0.70	2.45 ± 0.94^a^	2.21 ± 0.72^ab^	2.66 ± 1.24^ac^	20.16	< 0.001

*Abnormal distribution; NTNW, normal triglyceride normal waist; HTG, hypertriglyceridemia; EW, enlarged waist; HTW, hypertriglyceridemia-waist; ^a^ compare with NTNW group, *P* < 0.05; ^b^ compare with HTG group, *P* < 0.05; ^c^ compare with EW group, *P* < 0.05.

Further analysis revealed that there were differences in the basic characteristics of both boys and girls across phenotypes (all *P* < 0.01). The boys with the HTW phenotype had higher weight-Zscore, BMI, BMI-Zscore, WC, WC-Zscore, WHtR, SBP, SBP-Zscore, DBP, DBP-Zscore, TG and LDL-C than those with the NTNW, HTG and EW phenotypes respectively (*P* < 0.05). And weight-Zscore, BMI, BMI-Zscore, WC, WC-Zscore, WHtR, SBP, SBP-Zscore, DBP, DBP-Zscore, TG, and TC were all higher in girls with HTW phenotype than in girls with NTNW, HTG, and EW phenotypes, respectively (all *P* < 0.05), as shown in Tables 2 and 3.

**Table 2:** Comparison of basic characteristics of boys with different HTW phenotypes.

Variables	NTNW (N = 661)	HTG (N = 45)	EW (N = 149)	HTW (N = 48)	F/H	*P* value
Age (year)	15.0 ± 1.5	15.3 ± 1.5	14.7 ± 1.3^ab^	14.7 ± 1.6^b^	3.88	0.009
Height (cm)	170.2 ± 8.1	171.5 ± 7.2	174.0 ± 6.8^a^	173.6 ± 7.3^a^	11.29	< 0.001
Height-Z score	0.27 ± 0.92	0.38 ± 0.80	0.78 ± 0.73^ab^	0.80 ± 0.86^ab^	17.15	< 0.001
Weight (kg)	54.9 ± 8.0	55.4 ± 6.1	78.4 ± 11.8^ab^	81.9 ± 10.3^abc^	394.16	< 0.001
Weight-Z score	− 0.27 ± 0.64	− 0.27 ± 0.49	1.61 ± 0.87^ab^	2.00 ± 0.70^abc^	442.87	< 0.001
BMI (kg/m^2^)	18.9 ± 2.0	18.9 ± 2.0	25.9 ± 3.1^ab^	27.2 ± 2.8^abc^	542.36	< 0.001
BMI-Z score	− 0.45 ± 0.56	− 0.47 ± 0.56	1.45 ± 0.84^ab^	1.86 ± 0.74^abc^	537.38	< 0.001
WC (cm)	68.9 ± 4.7	70.5 ± 4.4	89.2 ± 8.9^ab^	92.7 ± 9.0^abc^	661.11	< 0.001
WC-Z score	− 0.52 ± 0.48	− 0.39 ± 0.46	1.54 ± 0.85^ab^	1.98 ± 0.84^abc^	725.95	< 0.001
WHtR	0.41 ± 0.03	0.41 ± 0.03	0.51 ± 0.05^ab^	0.53 ± 0.06^abc^	618.03	< 0.001
SBP (mm Hg)	111.1 ± 10.6	111.9 ± 12.7	121.0 ± 10.1^ab^	124.4 ± 10.9^abc^	52.36	< 0.001
SBP-Z score	− 0.04 ± 0.94	0.02 ± 1.09	0.82 ± 0.90^ab^	1.14 ± 0.97^abc^	52.10	< 0.001
DBP (mm Hg)	65.9 ± 7.9	67.3 ± 7.7	69.5 ± 7.0^a^	74.2 ± 8.8^abc^	23.82	< 0.001
DBP-Z score	− 0.24 ± 0.97	− 0.09 ± 0.95	0.22 ± 0.86^ab^	0.83 ± 1.15^abc^	25.06	< 0.001
FPG (mmol/L)	4.76 ± 0.72	5.29 ± 1.21	4.83 ± 0.60^b^	4.84 ± 0.81^b^	7.59	< 0.001
TG (mmol/L)*	0.82 (0.66,1.02)	1.67 (1.57,1.84)^a^	0.97 (0.77,1.23)^ab^	1.96 (1.61,2.37)^abc^	277.30	< 0.001
TC (mmol/L)	3.86 ± 0.88	4.61 ± 1.11^a^	3.84 ± 0.82^b^	4.79 ± 0.95^ac^	24.21	< 0.001
HDL-C (mmol/L)	1.46 ± 0.36	1.59 ± 0.49^a^	1.24 ± 0.28^ab^	1.25 ± 0.35^ab^	23.07	< 0.001
LDL-C (mmol/L)	2.11 ± 0.71	2.49 ± 0.89^a^	2.25 ± 0.73^a^	2.90 ± 1.38^abc^	18.56	< 0.001

*Abnormal distribution; NTNW, normal triglyceride normal waist; HTG, hypertriglyceridemia; EW, enlarged waist; HTW, hypertriglyceridemia-waist; ^a^ compare with NTNW group, *P* < 0.05; ^b^ compare with HTG group, *P* < 0.05; ^c^ compare with EW group,*P* < 0.05.

**Table 3:** Comparison of basic characteristics of girls with different HTW phenotypes.

Variables	NTNW (N = 321)	HTG (N = 37)	EW (N = 179)	HTW (N = 38)	F/H	*P* value
Age (year)	14.3 ± 1.6	14.2 ± 1.5	14.8 ± 1.5^a^	14.4 ± 1.6	3.25	0.022
Height (cm)	161.1 ± 5.7	159.6 ± 6.5	164.8 ± 6.0^ab^	163.7 ± 6.0^ab^	18.62	< 0.001
Height-Z score	− 0.73 ± 0.77	− 0.90 ± 0.75	− 0.37 ± 0.82^ab^	− 0.42 ± 0.83^ab^	10.44	< 0.001
Weight (kg)	48.1 ± 4.9	48.7 ± 4.8	64.1 ± 8.8^ab^	66.1 ± 9.6^ab^	267.15	< 0.001
Weight-Z score	− 0.74 ± 0.38	− 0.66 ± 0.42	0.50 ± 0.74^a**b**^	0.73 ± 0.77^abc^	244.35	< 0.001
BMI (kg/m^2^)	18.6 ± 1.7	19.1 ± 1.9	23.6 ± 2.8^ab^	24.7 ± 3.6^abc^	237.21	< 0.001
BMI-Z score	− 0.52 ± 0.47	− 0.34 ± 0.57	0.85 ± 0.78^ab^	1.18 ± 0.93^abc^	234.64	< 0.001
WC (cm)	68.7 ± 3.8	69.9 ± 3.0	82.8 ± 6.9^ab^	85.1 ± 8.4^abc^	332.46	< 0.001
WC-Z score	− 0.49 ± 0.35	− 0.34 ± 0.30	0.91 ± 0.71^ab^	1.21 ± 0.83^abc^	343.02	< 0.001
WHtR	0.43 ± 0.03	0.44 ± 0.02^a^	0.50 ± 0.04^ab^	0.52 ± 0.05^abc^	249.56	< 0.001
SBP (mm Hg)	106.8 ± 9.2	105.0 ± 7.8	112.2 ± 10.6^ab^	117.2 ± 11.4^abc^	22.56	< 0.001
SBP-Z score	− 0.47 ± 0.81	− 0.62 ± 0.71	0.03 ± 0.93^ab^	0.46 ± 0.98^abc^	24.51	< 0.001
DBP (mm Hg)	68.1 ± 7.3	67.2 ± 5.7	70.4 ± 8.3^ab^	73.4 ± 8.8^abc^	8.44	< 0.001
DBP-Z score	0.03 ± 0.93	− 0.09 ± 0.72	0.32 ± 1.03^ab^	0.67 ± 1.09^abc^	7.97	< 0.001
FPG (mmol/L)	4.73 ± 0.59	5.00 ± 0.97^a^	4.63 ± 0.47^b^	4.81 ± 0.49	4.63	0.003
TG (mmol/L)*	0.86 (0.69,1.03)	1.67 (1.53,1.96)^a^	0.90 (0.75,1.27)^ab^	1.81 (1.55,2.15)^abc^	203.94	< 0.001
TC (mmol/L)	3.91 ± 0.95	4.69 ± 1.46^a^	4.00 ± 0.82^b^	4.37 ± 1.12^abc^	8.92	< 0.001
HDL-C (mmol/L)	1.52 ± 0.42	1.71 ± 0.73^a^	1.43 ± 0.33^ab^	1.33 ± 0.40^ab^	6.90	< 0.001
LDL-C (mmol/L)	2.03 ± 0.68	2.40 ± 1.01^a^	2.17 ± 0.71^a^	2.36 ± 0.97^a^	5.02	0.002

*Abnormal distribution; NTNW, normal triglyceride normal waist; HTG, hypertriglyceridemia; EW, enlarged waist; HTW, hypertriglyceridemia-waist; ^a^ compare with NTNW group, *P* < 0.05; ^b^ compare with HTG group, *P* < 0.05; ^c^ compare with EW group,*P* < 0.05.

### Prevalence of cardiovascular risk factors and cardiovascular risk factors clustering in the participants across different HTW phenotype groups

**Figure 1:**
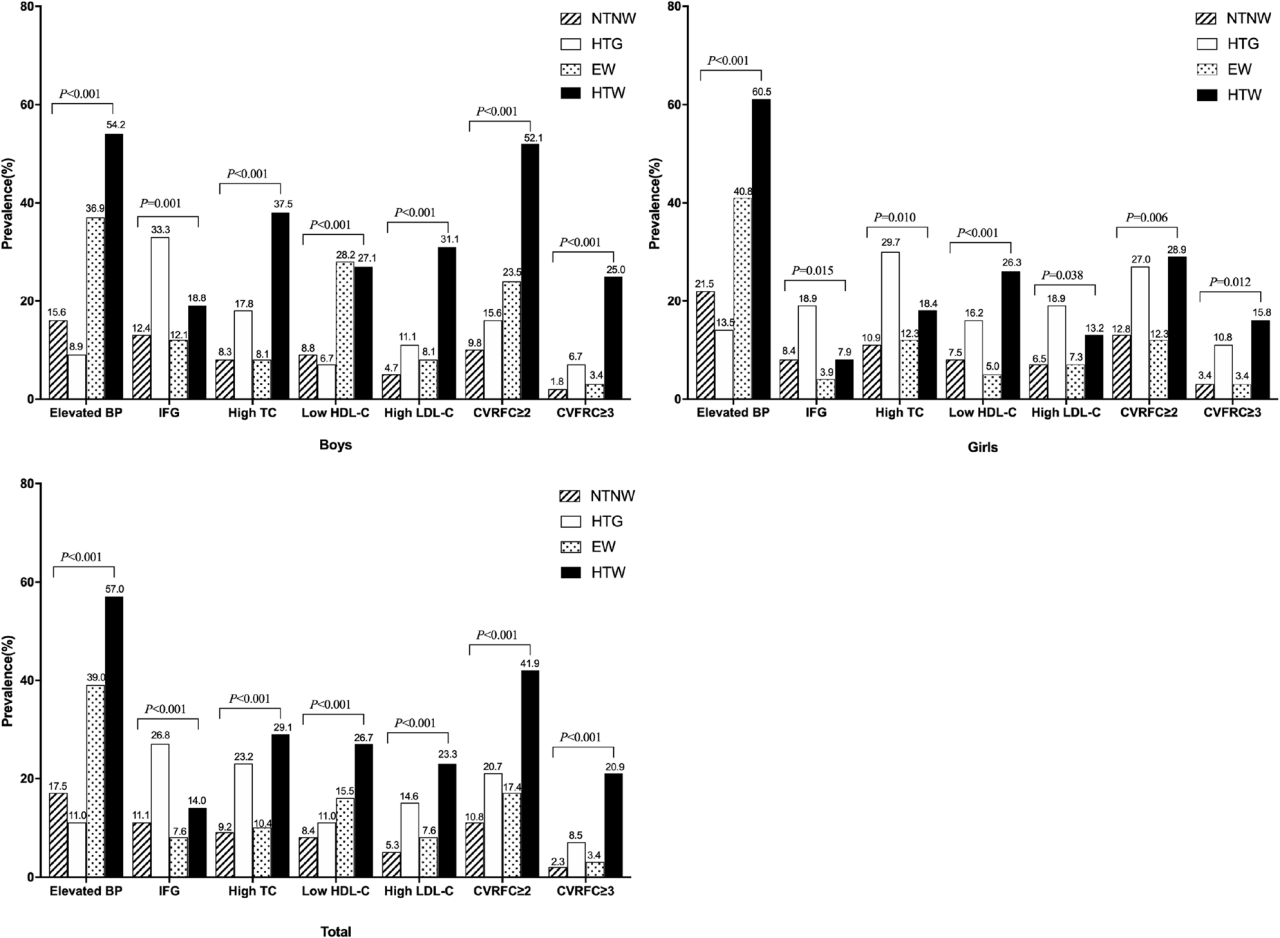
Prevalence of cardiovascular risk factors in the participants across different HTW phenotype groups. *Note*: IFG, impaired fasting glucose; NTNW, normal triglyceride normal waist; HTG, hypertriglyceridemia; EW, enlarged waist; HTW, hypertriglyceridemia-waist; CVRFC, cardiovascular risk factor clustering.

Figure 1 shows that the prevalence of elevated BP, IFG, high TC, low HDL-C, high LDL-C, CVRFC ≥ 2 and CVRFC ≥ 3 differed by different HTW phenotypes in the total population and both boys and girls (all *P* < 0.05).

### Associations between different HTW phenotypes and cardiovascular risk factors clustering

Table 4 presents the results of binary logistic regression analyses for different HTW phenotypes with specific cardiovascular risk factors adjusted for BMI, gender and age. After adjustment for covariates, the HTW phenotype had a higher risk of Elevated BP, High TC and Low HDL-C compared to the NTNW phenotype, the OR and 95% CI were 2.71 (1.50–4.91), 2.26 (1.08–4.71) and 2.69 (1.27–5.71). After gender stratification, the results showed that compared with the NTNW phenotype, the HTW phenotype was a risk factor for Elevated BP, High TC, Low HDL-C and High LDL-C in boys, respectively, the HTG phenotype was a risk factor for IFG, High TC and Low HDL-C in girls, while EW phenotype was negatively associated with IFG (all *P* < 0.05).

**Table 4:** Binary Logistic regression analysis of different HTW phenotypes and cardiovascular risk factors.

Variables	Total^a^		Boys^b^		Girls^b^	
	OR (95%CI)	*P* value	OR (95%CI)	*P* value	OR (95%CI)	*P* value
**Elevated BP**						
NTNW	Reference		Reference		Reference	
HTG	0.53 (0.26–1.08)	0.081	0.55 (0.19–1.57)	0.264	0.50 (0.19–1.35)	0.172
EW	1.48 (0.97–2.25)	0.067	1.74 (0.93–3.28)	0.085	1.22 (0.69–2.17)	0.439
HTW	2.71 (1.50–4.91)	0.001	3.22 (1.39–7.42)	0.006	2.30 (0.98–5.41)	0.056
**IFG**						
NTNW	Reference		Reference		Reference	
HTG	3.27 (1.88–5.69)	< 0.001	3.40 (1.72–6.71)	< 0.001	2.81 (1.09–7.28)	0.033
EW	0.60 (0.31–1.15)	0.124	1.15 (0.50–2.62)	0.745	0.20 (0.06–0.65)	0.022
HTW	1.10 (0.46–2.61)	0.827	1.89 (0.65–5.48)	0.239	0.44 (0.09–2.18)	0.311
**High TC**						
NTNW	Reference		Reference		Reference	
**HTG**	2.96 (1.66–5.26)	< 0.001	2.24 (0.98–5.12)	0.056	3.97 (1.72–9.15)	0.001
EW	0.61 (0.33–1.12)	0.109	0.81 (0.31–2.13)	0.675	0.48 (0.21–1.08)	0.074
HTW	2.26 (1.08–4.71)	0.030	5.54 (1.96–15.67)	0.001	0.79 (0.24–2.54)	0.688
**Low HDL-C**						
NTNW	Reference		Reference		Reference	
HTG	1.49 (0.71–3.12)	0.295	0.79 (0.24–2.65)	0.704	2.26 (0.83–6.16)	0.111
EW	1.61 (0.90–2.88)	0.107	3.10 (1.49–6.44)	0.002	0.58 (0.20–1.64)	0.302
HTW	2.69 (1.27–5.71)	0.010	2.78 (1.04–7.39)	0.041	3.37 (1.02–11.10)	0.046
**High LDL-C**						
NTNW	Reference		Reference		Reference	
HTG	3.02 (1.51–6.02)	0.002	2.38 (0.87–6.56)	0.093	3.70 (1.40–9.82)	0.008
EW	0.63 (0.30–1.30)	0.209	0.99 (0.34–2.88)	0.990	0.43 (0.16–1.20)	0.107
HTW	2.21 (0.96–5.11)	0.063	4.80 (1.54–14.96)	0.007	0.84 (0.21–3.35)	0.805

^a^Adjusted BMI, gender and age; ^b^ adjusted BMI and age.

Table 5 indicates that before adjusting for variables, the HTW phenotype increased the risk of CVRFC ≥ 2 and CVRFC ≥ 3 compared to the NTNW phenotype. This risk persisted after adjusting for BMI, gender and age, OR and 95% CI were 2.40 (1.23–4.58) and 3.63 (1.49–8.86), respectively. After stratification by gender, similar results were found in boys, girls with the HTG phenotype had a higher risk of CVRFC ≥ 2, however, those with the EW phenotype had a lower risk of CVRFC ≥ 2 and CVRFC ≥ 3 compared with the NTNW phenotype (all *P* < 0.05).

**Table 5:** Binary Logistic regression analysis of different HTW phenotypes and cardiovascular risk factors clustering.

Variables	CVRFC ≥ 2				CVRFC ≥ 3			
	Model 1		Model 2		Model 1		Model 2	
	OR (95%CI)	*P *value	OR (95%CI)	*P *value	OR (95%CI)	*P *value	OR (95%CI)	*P* value
**Total**								
NTNW	Reference		Reference		Reference		Reference	
HTG	2.16 (1.22–3.83)	0.008	2.13 (1.19–3.81)	0.011	3.89 (1.62–9.36)	0.002	2.85 (1.03–7.94)	0.045
EW	1.74 (1.23–2.47)	0.002	0.75 (0.44–1.28)	0.298	1.45 (0.70–3.00)	0.321	0.40 (0.14–1.14)	0.086
HTW	5.95 (3.71–9.55)	< 0.001	2.40 (1.23–4.58)	0.010	11.04 (5.68–21.44)	< 0.001	3.63 (1.49–8.86)	0.005
**Boys**								
NTNW	Reference		Reference		Reference		Reference	
HTG	1.69 (0.73–3.94)	0.225	1.62 (0.69–3.81)	0.265	3.86 (1.05–14.22)	0.042	3.63 (0.97–13.53)	0.055
EW	2.82 (1.78–4.45)	< 0.001	1.53 (0.73–3.19)	0.258	1.88 (0.65–5.41)	0.243	0.82 (0.17–3.88)	0.804
HTW	9.97 (5.35–18.55)	< 0.001	4.98 (2.03–12.25)	< 0.001	18.03 (7.57–42.93)	< 0.001	7.22 (1.67–31.22)	0.008
**Girls**								
NTNW	Reference		Reference		Reference		Reference	
HTG	2.53 (1.14–5.61)	0.022	2.64 (1.16–6.02)	0.021	3.42 (1.03–11.33)	0.045	3.26 (0.96–11.08)	0.058
EW	0.96 (0.55–1.67)	0.876	0.38 (0.17–0.83)	0.015	0.98 (0.36–2.69)	0.965	0.24 (0.57–0.98)	0.047
HTW	2.78 (1.28–6.03)	< 0.001	1.11 (0.39–3.13)	0.845	5.38 (1.83–15.24)	0.002	1.12 (0.24–5.27)	0.886

Model 1, not adjusted; Model 2, adjusted for BMI and age (add a gender adjustment to the total).

### Comparison of the area under the ROC curve of different indicators and cardiovascular risk factors clustering

Table 6 shows the results of the ROC curve analysis. TG combining WC performed better than BMI, WHtR, TG or WC alone in detecting cardiovascular risk factor clustering. The AUC was 0.698 (0.661–0.736) and 0.782 (0.725–0.840) in predicting CVRFC ≥ 2 and CVRFC ≥ 3 after adjustment for BMI, gender and age. Similar results were found after gender stratification (all *P* < 0.01).

**Table 6:** Comparison of the area under the ROC curve of different indicators and cardiovascular risk factors clustering.

Variables	Total^a^			Boys^b^			Girls^b^		
	AUC	95%CI	*P* value	AUC	95%CI	*P* value	AUC	95%CI	*P* value
**CVRFC ≥ 2**									
BMI	0.663	(0.624–0.703)	< 0.001	0.688	(0.637–0.739)	< 0.001	0.658	(0.600–0.715)	< 0.001
WHtR	0.665	(0.626–0.705)	< 0.001	0.687	(0.636–0.739)	< 0.001	0.657	(0.600–0.714)	< 0.001
TG	0.697	(0.659–0.734)	< 0.001	0.706	(0.657–0.756)	< 0.001	0.715	(0.662–0.769)	< 0.001
WC	0.664	(0.625–0.703)	< 0.001	0.689	(0.638–0.741)	< 0.001	0.662	(0.604–0.720)	< 0.001
TG combining WC	0.698	(0.661–0.736)	< 0.001	0.706	(0.657–0.756)	< 0.001	0.714	(0.661–0.768)	< 0.001
**CVRFC ≥ 3**									
BMI	0.711	(0.641–0.781)	< 0.001	0.734	(0.631–0.837)	< 0.001	0.648	(0.545–0.750)	0.010
WHtR	0.714	(0.644–0.784)	< 0.001	0.735	(0.632–0.838)	< 0.001	0.654	(0.554–0.754)	0.007
TG	0.779	(0.720–0.838)	< 0.001	0.790	(0.703–0.878)	< 0.001	0.752	(0.671–0.834)	< 0.001
WC	0.712	(0.643–0.782)	< 0.001	0.736	(0.635–0.837)	< 0.001	0.646	(0.542–0.751)	0.011
TG combining WC	0.782	(0.725–0.840)	< 0.001	0.790	(0.704–0.877)	< 0.001	0.755	(0.679–0.832)	< 0.001

^a^Adjusted BMI gender and age; ^b^ adjusted BMI and age.

## Discussion

In this study, we found that after adjusting for BMI, gender and age, the HTW phenotype had a higher risk of CVRFs compared to the NTNW phenotype. A one-year cohort study of children and adolescents showed that the HTW phenotype was a risk factor for longitudinal changes in SBP during follow-up^17^. Another study showed that the HTW phenotype was a strong predictor of incident hypertension, those with HTW phenotype were 2.3 times more likely to develop hypertension than those with NTNW phenotype after adjusting for gender and age^18^. The results of other studies also suggest that the HTW phenotype with a higher prevalence of hypertension compared to the NTNW phenotype^11^. Those are consistent with the results of the present study. Esmaillzadeh et al. suggest that adolescents with the HTW phenotype are not significantly associated with the development of IFG compared to adolescents with the NTNW phenotype^19^. However, another study of children and adolescents identified that after adjusting for confounding variables, an increase in fasting glucose means of 3.87 mg/dl (95%CI: 1.68–6.05) at one-year follow-up in those with the HTW phenotype^17^. Several studies in adults have also shown that the HTW phenotype is associated with IFG and even with the incident of type 2 diabetes^9,20,21^. Our study showed that compared with the NTNW phenotype, the HTW phenotype was not associated with IFG in both boys and girls, but the EW phenotype was negatively associated with IFG in girls after adjusting for BMI and age. We also found that compared with the NTNW phenotype, the girls with the EW phenotype had lower FPG ​​and the prevalence of IFG. This difference may be related to differences in study populations and regions, as well as the lower prevalence of IFG in adolescents in this population. Further explanation of the association between the HTW phenotype and glucose in adolescents is still needed in more studies.

The previous study illustrated that adolescents with the HTW phenotype were significantly more likely to have high TC (OR = 2.9; 95%CI:2.0–4.2), high LDL-C (OR = 1. 8; 95% CI 1.3–2.7) and low HDL-C (OR = 1.6; 95%CI:1.3–2.0) after controlling for potential confounding variables^19^. Another study on adolescents thought that after controlling for age and gender, adolescents with the high TG and high WHtR were more likely to have high TC (OR = 7.8; 95%CI:3.5–17.3), high LDL-C (OR = 9.4; 95%CI:2.8–31.2) and low HDL-C (OR = 10.8; 95% CI = 6.9–17.0) than those adolescents with normal TG and normal WHtR^22^. Other studies also showed that HTW was associated with high TC and low HDL-C in children and adolescents aged 10–18 years^23–25^. Adult studies have found that individuals with the HTW phenotype have a greater chance of having low HDL-C and LDL-C compared to individuals with the NTNW phenotype^11,26,27^. We obtained similar results in boys, but in girls, the HTW phenotype was associated with low HDL-C, but not with high TC and high LDL-C. This difference may be related to differences in gender and sex hormone levels, but more definitive underlying mechanisms need to be further investigated.

The clustering of CVRFs among adolescents is known to be associated with accelerated atherosclerosis and an increased risk of many chronic diseases, such as hypertension, hyperglycemia and dyslipidemia in adulthood^4,28^. Therefore, after confirming the association of the HTW phenotype with individual CVRFs, our study further analyzed its association with CVRFC and the results presented that the HTW phenotype was related to an increased risk of CVRFC ≥ 2 (OR = 2.40; 95%CI:1.23–4.58) and CVRFC ≥ 3 (OR = 3.63; 95%CI:1.49–8.86) in adolescents after adjustment for BMI, gender and age. Previous studies in adolescents have shown that the HTW phenotype is a stronger risk factor for CVRFC ≥ 1 (OR = 1.4; 95%CI:1.1–1.7) and CVRFC ≥ 2 (OR = 2.2; 95%CI:1.6–3.0) compared to adolescents with the NTNW phenotype after adjusting for potential confounding variables^19^. Bailey et al. found that in participants aged 10–19 years with HTW phenotype, the odds of having CVRFC ≥ 1 (OR = 4.78; 95%CI:1.32–17.29) and CVRFC ≥ 2 (OR = 7.16; 95%CI:2.38–21.54) were higher than those without the HTW phenotype^24^. This is similar to the results of our study. Another adult study showed that hypertensive adults with the HTW phenotype were significantly more likely to have all CVRFs compared to the NTNW group, and in particular for 8.35 times (95% CI 5. 92–11.79) more likely to have CVRFC ≥ 3^12^.

Regarding the HTW phenotype increased risk of cardiovascular risk may be associated with insulin resistance and endothelial dysfunction. First, the increase in WC, a proxy for abdominal fat, reflects some extent the accumulation of visceral and subcutaneous fat tissue. In the case of central obesity, visceral adipocytes release excess fatty acids and pro-inflammatory adipocytokines such as leptin and tumour necrosis factor-alpha into the portal circulation, leading to increased hepatic adiposity and insulin resistance, which further activates the renin–angiotensin–aldosterone system, increasing sympathetic activity, enhancing procoagulant activity, and inducing endothelial dysfunction, leading to hypertension and other cardiovascular diseases^29–31^. And a recent study found that high TG and high WC is a state of insulin resistance in adolescents^32^. Besides, a meta-analysis showed a significant correlation between the HTW phenotype and insulin resistance^20^. When the body has both abdominal obesity and high triglycerides, there may be a superimposed effect on insulin resistance. Insulin resistance has been identified as a major cause of increased cardiovascular risk factors^33^.

After stratification by gender, similar results were found in boys, however, girls with the EW phenotype had a lower risk of CVRFC ≥ 2 and CVRFC ≥ 3 compared with the NTNW phenotype when adjusting for BMI and age. Our further study analyzed that after adjustment for BMI and age, among adolescents with normal TG, the risk of IFG and CVRFC ≥ 2 in girls with enlarged WC were 0.17 times (95%CI:0.05–0.61, *P* = 0.007) and 0.36 times (95%CI:0.15–0.86, *P* = 0.022) lower than those girls with normal WC, respectively. Conversely, elevated TG levels increased the risk of IFG and CVRFC in girls with normal WC. It means when adjusted for the effect of BMI, it appeared to suggest that enlarged WC levels were protective against IFG and CVRFC in girls with normal TG. The results in girls were unexpected. But this explains why girls with the HTW phenotype did not have an increased risk of IFG and CVRFC compared with the NTNW phenotype. Data from a 6-year cohort study showed that FPG, TG, TC levels and the prevalence of T2DM decreased with increasing WC in females (*P* < 0.001)^34^. Another cohort study also showed that females with a WC gain had lower levels of TG and FPG at baseline than those without a gain (*P* < 0.05)^35^. WC serves as a simple surrogate for abdominal fat, which includes visceral fat tissue (VAT) and subcutaneous fat tissue (SAT). Data from McLaughlin et al. demonstrate that after adjustment for BMI and VAT, SAT is protective for insulin resistance, whereas VAT, after adjustment for SAT and BMI, has the opposite effect^36^. Studies have shown differences in TG lipolysis and turnover in visceral and subcutaneous white adipose tissue, and the proportion of VAT or SAT in abdominal fat determines the main factor of metabolic disorders, and these effects were more pronounced in girls than boys^37–39^. In addition, it may be related to testosterone (TT) and sex hormone-binding globulin (SHBG). In experimental studies, TT has been found to be a protective factor against atherosclerosis via suppressing pro-inflammatory cytokine activity and immuno-modulating effects^40^. A 5.7-year longitudinal study showed that the model comprised age, SBP, TC, WC, TT and SHBG, continuous values of SHBG significantly protected independently against diabetes risk in females (RR for 1-SD increment was 0.70[95%CI:0.59–0.83])^41^. But these are far from explaining this interesting phenomenon, and more large sample studies or studies on the mechanism are still needed.

Previous study results suggest that the HTW phenotype may be as discriminant as the NCEP-ATP III or the IDF criteria and could be used as an initial screening approach to identify individuals with deteriorated cardiometabolic risk markers^42^. Some studies of adolescents suggested HTW phenotype as a simple marker to identify adolescents at risk for metabolic syndrome (MetS) and other metabolic abnormalities^19,23^. Other adult studies have also shown that the HTW phenotype is independently associated with CVRFs and suggest that the HTW phenotype may be a simple and useful tool to screen individuals for future cardiovascular disease risk^9–11,43,44^. Liu et al. showed that the HTW phenotype is a reliable tool for identifying MetS, with an AUC of 0.843 (0.824–0.862) in men and 0.839 (0.813–0.865) in women^45^. Another study showed an AUC of 0.81 for TG*WC to predict metabolic syndrome^46^. In the present study, TG combining WC predicted AUC of 0.698 and 0.782 for CVRFC ≥ 2 and CVRFC ≥ 3 in adolescents respectively, which performed better than BMI, WHtR, TG or WC alone. Lee et al. also concluded that the combination of TG and WC has been illustrated as the best indicator of overall MetS in both genders^47^. These findings suggest that the HTW phenotype is not only strongly associated with individual cardiovascular risk factors, but also has important implications for the identification of metabolic syndromes and cardiovascular risk factor clustering.

In this study, we identified the important role of a simple combination of TG and WC in identifying cardiovascular risk factors and cardiovascular risk factors clustering in adolescents, and the protective effect of WC on IFG and cardiovascular risk factors clustering in girls with normal TG. This implies that for adolescents, a simple primary screening with TG and WC indicators can provide some basis for identifying early signs of cardiovascular disease. However, there are several limitations of the study that should be noted. Firstly, this cross-sectional study limits the causal interpretation of the observed associations. Secondly, this study did not assess some confounding factors such as lifestyle and physical activity, which may have influenced our results. Thirdly, regarding the protective effect of EW phenotype in girls on IFG and CVRFC and the confidence interval is wide in Table 5, which may be caused by the small sample size. Finally, the results cannot be generalized to other populations due to the age and ethnicity limitations of the participants. More cohort studies are needed in the future to explore the causal relationship, taking into account some confounding factors (e.g. diet, physical activity and family history of hypertension). This study only explored adolescents aged 12–18 years, and future studies could appropriately widen the age range or even examine whether there are differences between ages. Regarding the particular results in girls, this is a point well worth exploring in depth. In the discussion, we think that there may be an association to subcutaneous fat tissue (SAT), testosterone (TT) and sex hormone-binding globulin (SHBG) in which future studies could consider whether there is a mediating role for these factors.

## Conclusions

In conclusion, after adjustment for BMI and age, compared with the NTNW phenotype, boys with the HTW phenotype were at higher risk for cardiovascular risk factors and cardiovascular risk factors clustering, and girls with the EW phenotype had a lower risk of cardiovascular risk factors clustering. TG combining WC performed better than BMI, WHtR, TG or WC alone in predicting cardiovascular risk factor clustering.

## Data Availability

The datasets used and/or analyzed during the current study are not publicly available but are available from the corresponding author on reasonable request.
